# Antimicrobial resistance profile and multidrug resistance patterns of *Streptococcus pneumoniae* isolates from patients suspected of pneumococcal infections in Ethiopia

**DOI:** 10.1186/s12941-021-00432-z

**Published:** 2021-04-20

**Authors:** Bekele Sharew, Feleke Moges, Gizachew Yismaw, Wondwossen Abebe, Surafal Fentaw, Didrik Vestrheim, Belay Tessema

**Affiliations:** 1 Department of Medical Microbiology, School of Biomedical and Laboratory Sciences, College of Medicine and Health Sciences, University of Gondar, P.O. Box 196, Gondar, Ethiopia; 2 Department of Medical Laboratory Sciences, College of Medicine and Health Sciences, Wollo University, P. O. Box 45, Dessie, Ethiopia; 3 Ethiopian Public Health Institute, P. O. Box 641, Addis Ababa, Ethiopia; 4 Norwegian Institute of Public Health, Oslo, Norway

**Keywords:** Antimicrobial resistance, Multidrug resistance, *Streptococcus pneumoniae*

## Abstract

**Background:**

Antimicrobial-resistant strains of *Streptococcus pneumoniae* have become one of the greatest challenges to global public health today and inappropriate use of antibiotics and high level of antibiotic use is probably the main factor driving the emergence of resistance worldwide. The aim of this study is, therefore, to assess the antimicrobial resistance profiles and multidrug resistance patterns of *S. pneumoniae* isolates from patients suspected of pneumococcal infections in Ethiopia.

**Methods:**

A hospital-based prospective study was conducted from January 2018 to December 2019 at Addis Ababa city and Amhara National Region State Referral Hospitals. Antimicrobial resistance tests were performed from isolates of *S. pneumoniae* that were collected from pediatric and adult patients. Samples (cerebrospinal fluid, blood, sputum, eye discharge, ear discharge, and pleural and peritoneal fluids) from all collection sites were initially cultured on 5% sheep blood agar plates and incubated overnight at 37 °C in a 5% CO_2_ atmosphere. *Streptococcus pneumoniae* was identified and confirmed by typical colony morphology, alpha-hemolysis, Gram staining, optochin susceptibility, and bile solubility test. Drug resistance testing was performed using the E-test method according to recommendations of the Clinical and Laboratory Standards Institute.

**Results:**

Of the 57 isolates, 17.5% were fully resistant to penicillin. The corresponding value for both cefotaxime and ceftriaxone was 1.8%. Resistance rates to erythromycin, clindamycin, tetracycline, chloramphenicol and trimethoprim-sulfamethoxazole were 59.6%, 17.5%, 38.6%, 17.5 and 24.6%, respectively. Multidrug resistance (MDR) was seen in 33.3% isolates. The most common pattern was co-resistance to penicillin, erythromycin, clindamycin, and tetracycline.

**Conclusions:**

Most *S. pneumoniae* isolates were susceptible to ceftriaxone and cefotaxime. Penicillin has been used as a drug of choice for treating *S. pneumoniae* infection. However, antimicrobial resistance including multidrug resistance was observed to several commonly used antibiotics including penicillin. Hence, it is important to periodically monitor the antimicrobial resistance patterns to select empirical treatments for better management of pneumococcal infection.

## Introduction

*Streptococcus pneumoniae* is one of the leading causes of bacterial infections, ranging from self-limiting respiratory tract infections to severe invasive infections. It is a major public health concern, being responsible for an estimated 3.7 million episodes (2.7 million to 4.3 million) in children globally and approximately 50% of all pneumococcal deaths in 2015 occurred in four countries in Africa and Asia [1].

Antimicrobial resistance has been detected in all parts of the world; it is one of the greatest challenges to global public health today. The problem is increasing resistance to commonly used antimicrobial drugs which have elevated multidrug resistance (MDR). The fight against pneumococcal infections is based on curative treatment with antibiotics and preventive treatment using vaccination [2]. However, the emergence of resistant strains globally poses therapeutic problems. Nearly 40% of strains are resistant to penicillin, and penicillin resistance often correlates with resistance to other additional antibiotics such as macrolides, tetracyclines, etc. [3, 4].

In Asia, an alarming prevalence of penicillin and erythromycin-resistant *S. pneumoniae* has been described [5]. In 1995, an increase in the prevalence of resistance to penicillins, extended-spectrum cephalosporins, trimethoprim–sulfamethoxazole, and macrolides as well as MDR began to be recognized in Taiwan [6]. In Southern Finland, the proportion of MDR pneumococci doubled from 2007 to 2008, when it reached 3.6% [7].

Data on antimicrobial resistance from the African region are limited, but high-level penicillin-resistant *S. pneumoniae* have been described in central Africa [8]. The increasing trend of *S. pneumoniae* antimicrobial-resistance and the emergence of MDR isolates, which may result from inappropriate use of antibiotics and high level of antibiotic use is probably the main factor driving the emergence of resistance worldwide [9, 10].

In Ethiopia, there is lack of literatures on streptococcal drug resistance. A few studies have reported nasopharyngeal carriage rates [11, 12] and CSF sample sources [13, 14], for the presence of resistance to commonly prescribed antibiotics. But there is still a need for adequate baseline information for clinicians and other health professionals, and policy makers about the distribution of *S. pneumoniae* and to produce a structured data on drug resistance profile of this isolates in a regular base.

The present study aimed to investigate the antimicrobial resistance profile and multidrug resistance patterns of *S. pneumoniae* isolated from patients suspected of pneumococcal infections in Ethiopia using an E-test, where minimum inhibitory concentrations (MICs) determination is not routinely available.

## Materials and methods

### Study area, design, and period

 A hospital-based prospective study was conducted between January 2018 to December 2019 at Addis Ababa city (Yekatite12 Hospital, Alert Hospital, and International Clinical Laboratory) and Amhara National Regional State (ANRS) (University of Gondar Comprehensive Specialized Hospital, Felege Hiwot Comprehensive Specialized Hospital, and Dessie Regional Laboratory) Hospitals, Ethiopia. During the study period, 57 isolates were further confirmed as *S. pneumoniae* from clinical suspected patients. Suspected *S. pneumoniae* case means any reported case lacking confirmation of isolation from samples but has been diagnosed with *S. pneumoniae* by a clinician or other medical practitioner using sign and symptoms of the disease. The clinical sources of isolates were cerebrospinal fluid (CSF), blood, eye discharge, sputum, pleural fluid, ear discharge and peritoneal fluid.

### Isolation of *Streptococcus pneumoniae*

As part of routine service, samples (CSF, blood, sputum, eye discharge, ear discharge, and other body fluids) were initially cultured on 5% sheep blood agar plates. All growing isolates were stored at − 20 °C and shipped to the University of Gondar Microbiology Laboratory for identification through ice box. All isolates received from the collection site were frozen at − 80 °C until further testing. Isolates were recovered by plating onto 5% sheep blood and incubated overnight at 37 °C in a 5% CO_2_ atmosphere. *Streptococcus pneumoniae* was identified and confirmed by typical colony morphology, alpha-hemolysis, Gram staining, optochin susceptibility, and bile solubility test. After confirmation, the isolates were stocked in Skimmed Milk–Trypticase Soy–Glucose–Glycerol (STGG) medium and preserved at − 80 °C.

### *Streptococcus pneumoniae* antimicrobial susceptibility testing

The strains were shipped in dry ice to the Norwegian Reference laboratory for pneumococci at the Norwegian Institute of Public Health where antimicrobial susceptibility testing was performed. Minimum inhibitory concentrations (MICs) were determined by E-test on Mueller–Hinton agar supplemented with 5% sheep blood by the recommendation of the manufacturer (Biomerieux®). The antimicrobials tested were penicillin G, cefotaxime, ceftriaxone, erythromycin, clindamycin, tetracycline, chloramphenicol, trimethoprim/sulfamethoxazole, norfloxacin, and oxacillin. The Clinical and Laboratory Standards Institute (CLSI) 2020 clinical breakpoints for *S. pneumoniae* were applied to categorize isolates as susceptible, intermediate, or resistant [15]. The CLSI states that the 1-µg oxacillin disk diffusion test is an effective screening method commonly used in clinical laboratories for the detection of penicillin-resistant pneumococci.

The CLSI breakpoints for penicillin, cefotaxime, and ceftriaxone, differ between meningeal and non-meningeal infections. For penicillin, the breakpoints for meningitis is susceptible ≤ 0.06 µg/ml and resistant ≥ 0.12 µg/ml, and for non-meningitis ≤ 2 µg/ml susceptible, 4 µg/ml intermediate and ≥ 8 µg/ml resistance. For Cefotaxime and ceftriaxone, the non-meningitis breakpoint was used to classify isolates as susceptible (MIC ≤ 1 µg/ml), intermediate (MIC = 2 µg/ml) or resistant (MIC ≥ 4 µg/ml) and for meningitis susceptible (MIC ≤ 0.5 µg/ml), intermediate (MIC = 1 µg/ml) or resistant (MIC ≥ 2 µg/ml).

Interpretive criteria for norfloxacin are not given by the CLSI 2020. Thus, we used the breakpoints for norfloxacin obtained from the European Committee on Antimicrobial Susceptibility Testing (EUCAST) [16]. Norfloxacin disk diffusion test is an effective screening method for the detection of Moxifloxacin and Levofloxacin resistant pneumococci. MDR was defined as resistant to at least one agent in three or more antimicrobial classes. *S. pneumoniae* ATCC 49619 was used as the quality control strain and was included in each set of tests to ensure accurate results.

#### Data analysis

Data were entered and analyzed using the Statistical Package for the Social Science (version 20; SPSS Inc, Chicago, IL, USA). Discrete variables were expressed as percentages and proportions.

## Results

Of the 57 patients positive for *S. pneumoniae*, 33 (57.9%) were males. The age of the patients ranged from 38 days to 71 years; 37 (67.9%) were children less than 18 years old and 20 (35.1%) were adults. Among 57 *S. pneumoniae* strains recovered from different clinical specimens, the most common clinical sources were CSF 36.4% (n = 20), blood 20% (n = 12), eye discharge 18.2% (n = 10), sputum 15.8% (n = 10), pleural fluid 3.5% (n = 2), ear discharge 3.5% (n = 2) and peritoneal fluid 1.7% (n = 1). Thus, 35 (61.4%) of the isolates were from cases of invasive pneumococcal disease (IPD), recovered from a sterile body site (CSF, blood, peritoneal and pleural fluids) and 22 (38.6%) were non-IPD strains, recovered from sputum, eye discharge and ear discharge (Table 1).

**Table 1:** Patient characteristics and isolation of pneumococci at Addis Ababa city and Amhara National Region State Referral Hospitals, Ethiopia, 2018–2019

Characteristics	Pneumococcal isolates according to clinical significance		
	IPD (N = 35)	Non-IPD (N = 22)	Total (N = 57)
Gender			
Male	24 (68.6%)	9 (40.9%)	33 (57.9%)
Female	11 (31.4%)	13 (59.1%)	24 (42.1%)
Age (years)			
≤ 18	23 (65.7%)	14 (63.6%)	37 (64.9%)
> 18	12 (34.3%)	8 (36.4%)	20 (35.1%)
Residence			
Urban	23 (65.7%)	9 (40.9%)	42 (73.7%)
Rural	12 (34.3%)	13 (59.1%)	15 (26.3%)
Data collection site			
Addis Ababa city	11 (31.4%)	15 (68.2%)	26 (45.6%)
ANRS	24 (68.6%)	7 (31.8%)	31 (54.4%)
Disease on-set season			
January–March	7 (20%)	6 (27.3%)	13 (22.8%)
April–June	11(31.4%)	6 (27.3%)	17 (29.8%)
July–September	9 (25.7%)	6 (27.3%)	15 (26.3%)
October–December	8 (22.9%)	4 (18.1%)	12 (21.1%)

*IPD* Invasive pulmonary diseases, *N* Number, *ANRS* Amhara National Regional State

### Antimicrobial resistance

Majority (75.4%) of isolates were resistant to at least one of the antibiotics tested. Intermediately resistant and fully resistant rates to penicillin were 26.3 and 17.5%, respectively. The corresponding values for both cefotaxime and ceftriaxone were 3.5 and 1.8%, respectively. Resistance rates to erythromycin, clindamycin, tetracycline, chloramphenicol and trimethoprim-sulfamethoxazole were 59.6%, 17.5%, 38.6%, 17.5 and 24.6%, respectively (Table 2). Norfloxacin (3.5%) and oxacillin (38.6%) were screening methods for the detection of moxifloxacin and levofloxacin, and penicillin-resistant pneumococci, respectively.

**Table 2:** Categorization of antimicrobial resistance to 10 antimicrobial agents in 57 *Streptococcus pneumoniae* isolates at Addis Ababa city and Amhara National Region State Referral Hospitals, Ethiopia, 2018–2019

Antibiotics	Sensitive N(%)	Intermediate N(%)	Resistant N (%)
Penicillin G (PEN)	32 (56.1)	15 (26.3)	10 (17.5)
Cefotaxime (CTX)	54 (94.7)	2 (3.5)	1(1.8)
Ceftriaxone (CRO)	54 (94.7)	2 (3.5)	1(1.8)
Erythromycin (ERY)	23 (40.4)	0	34 (59.6)
Clindamycin (CD)	46 (80.7)	1(1.8)	10 (17.5)
Tetracycline (TET)	33 (57.9)	2 (3.5)	22 (38.6)
Chloramphenicol (CHL)	47 (82.5)	0	10 (17.5)
Trimethoprim-sulfamethoxazole (SXT)	43 (75.4)	0	14 (24.6)
Norfloxacin (NOR)	55 (96.5)	NA	2 (3.5)
Oxacillin (OXA)	35 (61.4)	NA	22 (38.6)

*NA* Not applicable, *N* Number

Of the 57 isolates, 16 (28.1%) were resistant to only one class of antimicrobial, 14 (24.6%) were resistant to two classes of antimicrobials, while 19 (33.3%) were resistant to three or more classes of antimicrobials; eight (14%) isolates were susceptible to all antimicrobials tested. Penicillin, erythromycin, clindamycin and tetracycline-resistant pneumococcus were usually also resistant to other antimicrobials. The most common MDR patterns were ERY + CD + TET (17.5%), PEN + ERY + TET (10.5%), followed by PEN + ERY + CD (7%), PEN + CD + TET (7%) and PEN + CTX + CRO + TET (7%) (Table 3).

**Table 3:** Resistance to two or more antimicrobial classes among *Streptococcus pneumoniae* isolates at Addis Ababa city and Amhara National Region State Referral Hospitals, Ethiopia, 2018–2019

Antibiotics	Number	Percentage
PEN + ERY	6	10.5
PEN + CD	4	7
PEN + TET	6	10.5
PEN + CHL	1	1.8
PEN + SXT	3	5.3
ERY + CD	10	17.5
ERY + TET	19	33.3
ERY + CHL	9	15.8
ERY + SXT	5	8.8
CD + TET	10	17.5
CD + SXT	2	3.5
TET + CHL	1	1.8
TET + SXT	5	8.8
CHL + SXT	1	1.8
PEN + CTX + CRO	1	1.8
PEN + ERY + CD	4	7
PEN + ERY + TET	6	10.5
PEN + ERY + CHL	1	1.8
PEN + ERY + SXT	1	1.8
PEN + CD + TET	4	7
PEN + CD + SXT	1	1.8
PEN + TET + CHL	1	1.8
PEN + TET + SXT	1	1.8
ERY + CD + TET	10	17.5
ERY + CD + SET	2	3.5
CD + TET + SXT	2	3.5
PEN + CTX + CRO + ERY	1	1.8
PEN + CTX + CRO + TET	4	7
PEN + ERY + CD + SXT	1	1.8
PEN + CD + TET + SXT	1	1.8
ERY + CD + TET + SXT	1	1.8
PEN + ERY + CD + TET + SXT	1	1.8
PEN + CTE + CRO + ERY + TET + CHL	1	1.8
Total MDR (Greater than or equal to 3)	19	33.3

*PEN* Penicillin, *CTX* Cefotaxime, *CRO* Ceftriaxone, *ERY* Erythromycin, *CD* Clindamycin, *TET* Tetracycline, *CHL* Chloramphenicol, *SXT* Trimethoprim–sulfamethoxazole

## Discussion

*Streptococcus pneumoniae* is responsible for many community infections, with the main ones being pneumonia and meningitis. Pneumococcus has developed increased resistance to multiple classes of antibiotics. Developing countries face significant problems of antimicrobial resistance with poor diagnostic facilities, unauthorized sale of antimicrobials, lack of appropriate functioning drug regulatory mechanisms, and non-human use of antimicrobials such as in animal production [17, 18]. In this work, we investigated the antimicrobial resistance profile and MDR pattern of *S. pneumoniae* from clinical isolates in two regions in Ethiopia. Currently, the antibiotic resistance patterns of *S. pneumoniae* isolates vary widely from one country to another, but many studies are reporting a high frequency of antimicrobial resistance among pneumococcal isolates.

In the present study, the resistance rate of pneumococci to a penicillin (17.5%) was higher than other beta-lactam antibiotics such as cefotaxime (1.8%) and ceftriaxone (1.8%). Cefotaxime and ceftriaxone have a similar group of the spectrum. The resistance rate to penicillin in the present study was higher than studies in Central Africa (< 6%) [8], Tunisia (1.2%) [19] and the United States (14.8%) [9], but higher penicillin resistance rates were observed in Taiwan (43.3–73.2%) [20], Canada (26.1%) [21], China (88.3%) [22], Russia (28%) [23], Nigeria (28%) [24] and Guinea (21.5%) [25]. The high prevalence of penicillin resistance in this report implies that the use of penicillin for empiric treatment of suspected pneumococcal infection should no longer be recommended. However, cefotaxime and ceftriaxone show very good activities against *S. pneumoniae* which might be due to their lack of availability and high cost compared to commonly used penicillin [24].

The overall resistance rate of *S. pneumoniae* to erythromycin, clindamycin, tetracycline, chloramphenicol, and trimethoprim/sulfamethoxazole was 59.6%, 17.5%, 38.6%, 17.5%, and 24.6%, respectively. The rate of resistance to erythromycin was lower than studies done in Canada (100%) [21] and China (95.2%) [22], but higher than in Russia (26%) [23], Mozambique (23.6%) [26] and Pakistan (up to 29.7%) [27]. Seventeen and a half percent (17.5%) isolates were resistant to clindamycin. Higher clindamycin resistance rates were observed in Canada (40.6%) [21] and China (95.8%) [22]. In addition, 38.6% of our isolates were resistant to tetracycline, which is lower than studies in China (93.6%) [22] and Nigeria (73.5%) [24]. Furthermore, the resistance rate to trimethoprim/sulfamethoxazole was 24.6% in our study. Higher resistance rates were reported in Central Africa (up to 69%) [8], Canada (34.5%) [21], China (66.7%) [22], Russia (57%) [23], Nigeria (96.2%) [24] and Pakistan (86.6%) [27]. In addition, 17.5%% of our strains were resistant to chloramphenicol, which is comparable to 18.9% in Central Africa [9], but lower than in Niger where up to 60% was reported [24].

These differences in antimicrobial resistance may be explained by differences in the source of the isolates, geographical variability, high prevalence of HIV infection, and antibiotics usage. Higher rates of antibiotic resistance have been linked to high antimicrobial consuming countries [10]. In Ethiopia, antibiotics can be bought without a prescription and this probably leads to overuse and misuse of antibiotics which can promote the widespread antibiotic resistance strain in the area [28].

The emergence of MDR *S. pneumoniae* isolates has been a worldwide public health concern for several years. In this study, we demonstrated that penicillin-resistant *S. pneumoniae* strains were also resistant to erythromycin, tetracycline, and clindamycin (10.5%, 10.5%, and 7% of isolates, respectively). Erythromycin resistant *S. pneumoniae* strains were resistant to tetracycline, clindamycin, and chloramphenicol (33.3%, 17.5%, and 15.8% of isolates, respectively).

The most common MDR phenotype was resistance to penicillin, erythromycin, tetracycline, and clindamycin, detected in 33.3% of the total isolates. Such MDR rates were higher than those observed in China (21.4%) [22], Russia (22%) [23] and Portugal (26%) [29]. However, it was lower than studies reported from Tunisia (96.5%) [19], Nigeria (53.8%) [24] and China (99.4%) [30]. Moreover, the results reveal a common pattern of co-resistance to penicillin, erythromycin, clindamycin, and tetracycline. The empirical treatment of pneumococcal infections, especially invasive infections, often requires a combination of two or more antibiotics and longer durations. These conditions inevitably lead to the initiation of high or multidrug resistance among patients with infections. The limitation of this study was the small sample size, and also absence of data on the prior use of antibiotics, which could potentially bias the pneumococcal collection regarding frequency of antimicrobial resistant isolates. This could potentially restrict the scope of our findings not making them general for the rest of the country. This study has also some strength which providing a useful information that may be used to guide public health measures to contain antimicrobial resistance and pneumococcal disease.

## Conclusions

In the present study, most bacterial isolates were susceptible to ceftriaxone and cefotaxime. Penicillin has been used as a drug of choice for treating *S. pneumoniae* infection. However, antimicrobial resistance including multidrug resistance was observed to several commonly used antibiotics including penicillin. Hence, it is important to periodically monitor the antibiotic resistance patterns to choose empirical treatments for better management of pneumococcal infection. We recommend developing an action plan for the promotion of rational use of antimicrobials, strengthening of antimicrobial resistance surveillance, and implementation of an antimicrobial stewardship program.

## Data Availability

All data generated or analyzed during this study are included in this published article.

## Abbreviations

CLSI: Clinical and Laboratory Standards Institute
CSF: Cerebrospinal fluid
EUCAST: European Committee on Antimicrobial Susceptibility Testing
IPD: Invasive pneumococcal diseases
MDR: Multi-drug resistance
MICs: Minimum inhibitory concentration
PCV: Pneumococcal conjugate vaccine
STGG: Skimmed Milk–Trypticase Soy–Glucose–Glycerol
*S. Pneumoniae*: *Streptococcus pneumoniae*
WGS: Whole genome sequencing
